# Peroxisome Proliferator-Activated Receptor Activation is Associated with Altered Plasma One-Carbon Metabolites and B-Vitamin Status in Rats

**DOI:** 10.3390/nu8010026

**Published:** 2016-01-05

**Authors:** Vegard Lysne, Elin Strand, Gard F. T. Svingen, Bodil Bjørndal, Eva R. Pedersen, Øivind Midttun, Thomas Olsen, Per M. Ueland, Rolf K. Berge, Ottar Nygård

**Affiliations:** 1 Department of Clinical Science, University of Bergen, 5020 Bergen, Norway; elin.strand@uib.no (E.S.); gard.frodahl.tveitevag.svingen@helse-bergen.no (G.F.T.S.); Bodil.Bjorndal@k2.uib.no (B.B.); eva.pedersen@k2.uib.no (E.R.P.); olsen.thomas89@gmail.com (T.O.); per.ueland@ikb.uib.no (P.M.U.); Rolf.Berge@uib.no (R.K.B.); Ottar.Nygard@helse-bergen.no (O.N.); 2 Department of Heart Disease, Haukeland University Hospital, 5021 Bergen, Norway; 3 Bevital AS, 5021 Bergen, Norway; Bjorn.Midttun@uib.no; 4 Laboratory of Clinical Biochemistry, Haukeland University Hospital, 5021 Bergen, Norway; 5 KG Jebsen Centre for Diabetes Research, University of Bergen, 5009 Bergen, Norway

**Keywords:** dimethylglycine, methylmalonic acid, one-carbon metabolism, peroxisome proliferator-activated receptors, tetradecylthioacetic acid

## Abstract

Plasma concentrations of metabolites along the choline oxidation pathway have been linked to increased risk of major lifestyle diseases, and peroxisome proliferator-activated receptors (PPARs) have been suggested to be involved in the regulation of key enzymes along this pathway. In this study, we investigated the effect of PPAR activation on circulating and urinary one-carbon metabolites as well as markers of B-vitamin status. Male Wistar rats (*n* = 20) received for 50 weeks either a high-fat control diet or a high-fat diet with tetradecylthioacetic acid (TTA), a modified fatty acid and pan-PPAR agonist with high affinity towards PPARα. Hepatic gene expression of PPARα, PPARβ/δ and the enzymes involved in the choline oxidation pathway were analyzed and concentrations of metabolites were analyzed in plasma and urine. TTA treatment altered most biomarkers, and the largest effect sizes were observed for plasma concentrations of dimethylglycine, nicotinamide, methylnicotinamide, methylmalonic acid and pyridoxal, which were all higher in the TTA group (all *p* < 0.01). Hepatic *Pparα* mRNA was increased after TTA treatment, but genes of the choline oxidation pathway were not affected. Long-term TTA treatment was associated with pronounced alterations on the plasma and urinary concentrations of metabolites related to one-carbon metabolism and B-vitamin status in rats.

## 1. Introduction

Elevated plasma total homocysteine (tHcy) is related to increased risk of atherothrombotic cardiovascular disease (CVD) [1]. However, lowering of tHcy with B-vitamins has not improved prognosis among CVD patients [2], which is questioning a causal relationship and encourages investigation into novel mechanisms associated with elevated plasma tHcy [3]. Circulating and urinary concentrations of various metabolites along the choline oxidation pathway, which is linked to remethylation of Hcy, have been related to major lifestyle diseases including CVD and diabetes [4,5,6,7,8]. We have recently shown that higher plasma dimethylglycine (DMG) concentrations are associated with increased risk of acute myocardial infarction as well as total and cardiovascular mortality, independent of traditional risk markers including elevated plasma tHcy [6,7].

**Figure 1:**
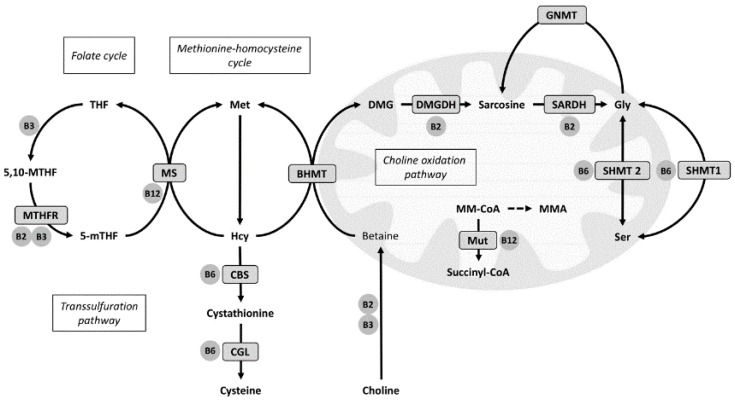
Overview of one-carbon metabolism related pathways. 5-mTHF indicates methyltetrahydrofolate; 5,10-MTHF, methylenetetrahydrofolate; BHMT, betaine-homocysteine methyltransferase; CBS, cystathionine β-synthase; CGL, cystathionine-γ-lyase; DMG, dimethylglycine; DMGDH, dimethylglycine dehydrogenase; Gly, glycine; GNMT, glycine N-methyltransferase; Hcy, homocysteine; Met, methionine; MMA, methylmalonic acid; MM-CoA, methylmalonyl CoA; MS, methionine synthase; MTHFR, methylenetetrahydrofolate reductase; Mut, methylmalonyl-CoA mutase; Sarc, sarcosine; SARDH, sarcosine dehydrogenase; Ser, serine; SHMT, serine-hydroxy-methyltransferase; THF, tetrahydrofolate.

Homocysteine (Hcy) resides at a branch point of three metabolic pathways. Remethylation of Hcy back to methionine is catalyzed either by the cobalamin-dependent methionine synthase (MS, EC 2.1.1.13) or betaine-homocysteine methyltransferase (BHMT, EC 2.1.1.5), using 5-methyltetrahydrofolate (mTHF) or betaine as the methyl donor, respectively. Hcy catabolism to form cysteine is carried out by the vitamin B6 dependent transsulfuration pathway [9] (Figure 1). Hcy metabolism is linked to the choline oxidation pathway by BHMT, which demethylates betaine to form DMG [10]. DMG is further oxidized to sarcosine and glycine by two mitochondrial flavoenzymes, *i.e.*, DMG dehydrogenase (DMGDH, EC 1.5.8.4) and sarcosine dehydrogenase (SARDH, EC 1.5.8.3) [11]. Interestingly, increased flux through BHMT has also been associated with decreased DNA methylation of the promoter region of the peroxisome proliferator-activated receptor (PPAR) α gene in mice, resulting in increased gene expression of PPARα and its target genes [12]. In rats, activation of PPARα has been demonstrated to reduce the genetic transcription of DMGDH, SARDH and glycine N-methyltransferase (GNMT, EC 2.1.1.20), as well as both enzymes of the transsulfuration pathway [13]. This indicates a relationship between PPARα and these pathways, and hence, we previously suggested that the association between elevated plasma DMG and CVD risk may partly be related to enhanced endogenous PPARα activity [6,7].

PPARα is a key regulator of energy metabolism [14], with a large number of identified target genes [15]. PPARα is activated by dietary or endogenous fatty acids and their derivatives [16]. Tetradecylthioacetic acid (TTA) is a sulfur-containing fatty acid analogue with a high affinity towards PPARα [17], and we have previously demonstrated significant increases in PPARα target genes in the liver after TTA treatment, also accompanied by reduced plasma and hepatic lipid levels [18]. Although PPARα has been thoroughly explored according to its role in lipid and glucose metabolism, the relationship between PPARα and other metabolic pathways has only recently gained attention. Involvement in amino acid metabolism has been demonstrated [19,20], and fibrates, which are specific PPARα ligands have consistently been associated with elevated plasma tHcy [21], as well as being associated with elevated urinary output of choline, betaine and DMG [22,23], linking PPARα to one-carbon metabolism. In terms of the choline oxidation pathway, PPARα activation has in rodent models been associated with a reduction in *Dmgdh* and *Sardh* mRNA [13] and lower protein level of SARDH [24], and recently, long-term TTA treatment was associated with lower protein expression of BHMT, DMGDH and SARDH [25]. However, whether TTA treatment affects the related metabolites has yet to be explored. Also, activation of PPARα has been shown to increase the synthesis of vitamin B3 from tryptophan by regulating key enzymes in this pathway [26], but whether PPAR activation influences the status of other B-vitamins is uncertain.

The aim of the current study was to investigate how PPAR activation by TTA supplementation affected blood and urinary concentrations of components of the choline oxidation pathway and one-carbon metabolites, as well as systemic markers of B-vitamin status.

## 2. Materials and Methods

### 2.1. Animals and Diets

Male Wistar rats (*n* = 20), 8–10 weeks old on arrival and weight 260–270 g (Taconic Europe A/S, Lille Skensved, Denmark), were randomly allocated to receive either a high fat control diet (Control) with 25% fat (23% lard, 2% soybean oil, weight/weight) or a high fat diet supplemented with TTA (TTA) (22.6% lard, 2% soybean oil, 0.4% TTA, weight/weight). The diets had the same amounts of micronutrients, and the rats had free access to water and feed during the study period. The animals investigated were part of a larger study, and more detailed descriptions of this experiment and the composition of the diets have previously been published [27]. Feed intake was comparable between groups, but the TTA treated rats gained less weight as compared to Controls [28].

After 50 weeks, the animals were sacrificed under non-fasting conditions by anaesthetization with Isofluorane (Forane, Abbott Laboratories, Abbott Park, IL, USA) inhalation. Blood was drawn by cardiac puncture and collected in BD Vacutainer tubes containing EDTA (Becton-Dickinson, Plymouth, UK). Urine was collected directly from the urinary bladder.

### 2.2. Ethics Statement

The animal experiments complied with the Guidelines for the Care and Use of Experimental Animal use and the study protocols were approved by the Norwegian State Board for Biological Experiments with Living animals (“Forsøksdyrutvalget”, permit number 2005140).

### 2.3. Biochemical Analyses

With the exception of plasma cobalamin, which was measured in only seven rats per group due to limited amounts of plasma, all blood metabolites were analyzed in 10 rats per group. Urinary metabolites were analyzed in 9 control animals and in 8 TTA treated rats. All analyses were performed at Bevital A/S (Bevital, Bergen, Norway). In plasma, methylmalonic acid (MMA), tHcy, cystathionine, serine and glycine were analyzed by gas chromatography coupled with tandem mass spectrometry (GC-MS/MS) [29]. Plasma choline, betaine, DMG, methionine and cysteine, as well as all vitamin B2, B3, and B6 forms and metabolites were analyzed by liquid chromatography coupled with tandem mass spectrometry (LC-MS/MS) [30,31]. Plasma folate [32] and cobalamin [33] were measured by microbiological assays. In urine, cysteine, cystathionine, sarcosine, glycine, serine and MMA were measured by GC-MS/MS [29], and methionine, choline, betaine and DMG by LC-MS/MS [30].

### 2.4. Gene Expression Analysis

RNA was purified from frozen liver samples and cDNA was produced as previously described [34]. Using probes and primers from Applied Biosystems (Foster City, CA, USA), real-time PCR was performed with Sarstedt 384 well multiply-PCR Plates (Sarstedt Inc., Newton, NC, USA) on *Ppara* (Rn00566193), *Ppard* (Rn 00565707), *Bhmt* (Rn00578255_m1), *Dmgdh* (Rn00594751), *Sardh* (Rn00454657_m1) and *Gnmt* (Rn00567215_m1). Three reference genes were included: 18s (Kit-FAM-TAMRA (Reference RT-CKFT-18s)) from Eurogentec (Seraing, Belgium), glyceraldehyde-3-phosphate dehydrogenase (Gapdh, Mm99999915_g1) from Applied Biosystems, and ribosomal protein, large, P0 (Rplp0, Gene ID 11837) from Thermo Fisher Scientific Inc. (Waltham, MA, USA). The absolute quantification was normalized according to the reference genes as previously described [28], and the result is presented normalized to 18s, which was selected by the NormFinder algorithm which ranks the candidate reference genes according to their expression stability [35].

### 2.5. Statistical Analyses and Presentation of Data

The plasma concentrations of metabolites are presented as means (SD). Normality was assessed by the Kolmogorov-Smirnov test, and the groups were compared with independent samples *t*-tests. Standardized mean differences (SMD) (95% confidence interval) were calculated.

The concentrations of urinary metabolites were given as µmol of metabolite per mmol creatinine (SD) to correct for dilution. We evaluated the relationship between plasma and urinary concentrations of metabolites by calculating Pearson’s correlation coefficients.

Statistics were performed using IBM SPSS Statistics for Windows, version 21 (SPSS IBM., Chicago, IL, USA), Prism 6.0 (GraphPad Software, Inc., La Jolla, CA, USA) and Microsoft Excel 2010. *p*-values < 0.01 were considered statistical significant, according to the Benjamini and Hochberg method of controlling the false discovery rate [36].

For the gene expression analyses, normality was analyzed by the D’Agostino and Pearson omnibus normality test. The expression of *Ppara*, *Bhmt*, *Dmgdh*, *Sardh* and *Gnmt* mRNA was compared by *t*-test while the expression of *Ppard* mRNA was compared by Mann-Whitney U test.

## 3. Results

**Figure 2:**
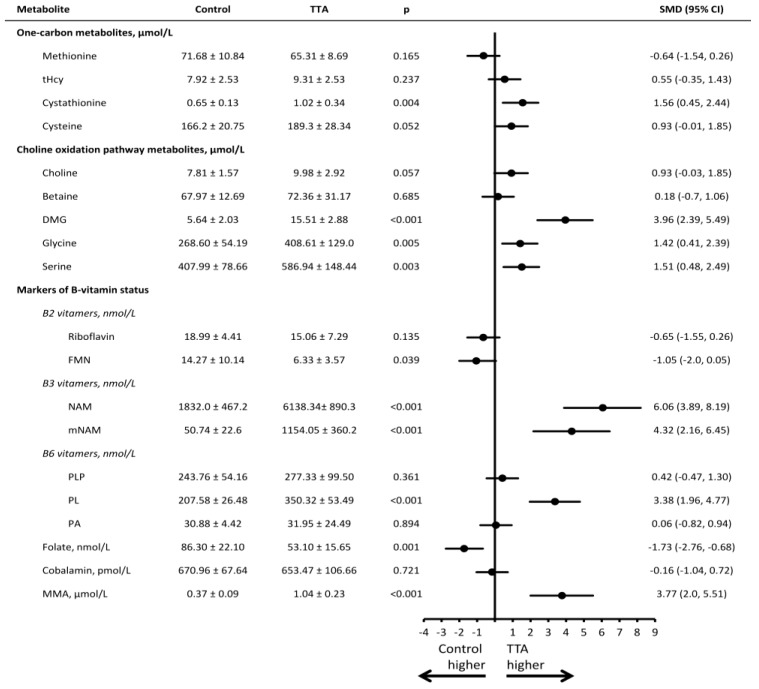
Plasma concentrations of metabolites in rats receiving a high fat control diet or a high fat diet with additional TTA treatment. Male Wistar rats were treated with a high-fat diet with or without additional TTA treatment, *n* = 10 in each group. Mean (SD) plasma concentration of the metabolites, as well as the standardized mean difference (95% CI) between the control and the TTA treated animals. DMG indicates dimethylglycine; FMN, flavin mononucleotide; MMA, methylmalonic acid; mNAM, N1-methylnicotinamide; NAM, nicotinamide; PA, pyridoxic acid; PL, pyridoxal; PLP, pyridoxal-5’-phosphate; SMD, standardized mean difference; tHcy, total homocysteine; TTA, tetradecylthioacetic acid.

**Figure 3:**
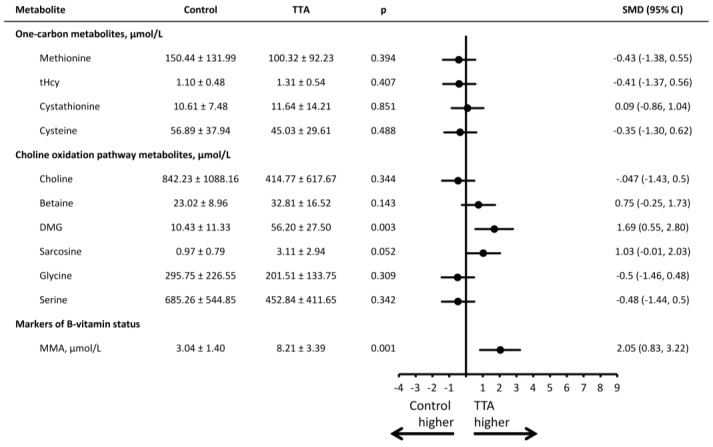
Urinary concentrations of metabolites in rats receiving a high fat control diet or a high fat diet with additional TTA treatment. Male Wistar rats were treated with a high-fat diet with or without additional TTA treatment, *n* = 10 in each group. Mean (SD) urinary concentration of the metabolites, as well as the standardized mean difference (95% CI) between the control and the TTA treated animals. DMG indicates dimethylglycine; MMA, methylmalonic acid; SMD, standardized mean difference; tHcy, total homocysteine; TTA, tetradecylthioacetic acid.

Mean (SD) concentrations for all plasma and urinary metabolites and the SMD between groups are presented in Figure 2 and Figure 3. Compared to rats receiving the control diet, rats treated with TTA differed in most metabolites and markers of B-vitamin status.

In terms of the transsulfuration pathway, higher concentration of plasma cystathionine was observed (SMD = 1.56 [0.45–2.44], *p* = 0.004). Regarding components of the choline oxidation pathway, rats in the TTA intervention group had higher concentrations of plasma DMG (SMD = 3.96 [2.39–5.49], *p* < 0.001), glycine (SMD = 1.42 [0.41–2.39], *p* = 0.005) and serine (SMD = 1.51 [0.48–2.49], *p* = 0.003). In urine, the TTA intervention group had higher concentrations of DMG (SMD = 1.69 [0.55–2.80], *p* = 0.003).

Among B-vitamers and their respective metabolites, we observed higher concentrations of plasma nicotinamide (NAM) (SMD = 6.06 [3.89–8.19], *p* < 0.001), N1-methylnicotinamide (mNAM) (SMD = 4.32 [2.16–6.45], *p* < 0.001) and pyridoxal (PL) (SMD = 3.38 [1.96–4.77], *p* < 0.001) in the TTA treated rats as compared to controls, whereas plasma folate was lower (SMD = −1.73 [−2.76–−0.68], *p* = 0.001). Riboflavin (SMD = −0.65, [−1.55–0.26], *p* = 0.13) and FMN (SMD = −1.05 [−2.0–0.05], *p* = 0.039) tended to be lower after TTA treatment. No difference was observed for plasma cobalamin, albeit both plasma and urine concentrations of MMA were higher among TTA treated rats vs controls (SMD = 3.77 [2.0–5.51] and 2.05 [0.83–3.22], respectively, both *p* < 0.001).

As shown in Table 1, there were strong, positive correlations between plasma and urinary concentrations for betaine (*r* = 0.62), DMG (*r* = 0.79) and MMA (*r* = 0.73), all *p* < 0.01.

**Table 1:** Correlations between blood and urinary concentrations of metabolites. Male Wistar rats were treated with either a low-fat control diet or a high-fat diet with or without additional TTA treatment, *n* = 17. Pearson correlation coefficients were calculated between the plasma and urinary concentration of metabolites.

	*r* (95% CI)	*P*
Methionine	−0.02	0.93
tHcy	−0.10	0.71
Cystathionine	0.25	0.33
Cysteine	0.09	0.73
Choline	0.08	0.93
Betaine	0.62	0.008
DMG	0.79	<0.001
Glycine	−0.13	0.63
Serine	−0.17	0.51
MMA	0.73	<0.001

DMG indicates dimethylglycine; MMA, methylmalnic acid; tHcy, ttal hmcysteine.

**Figure 4:**
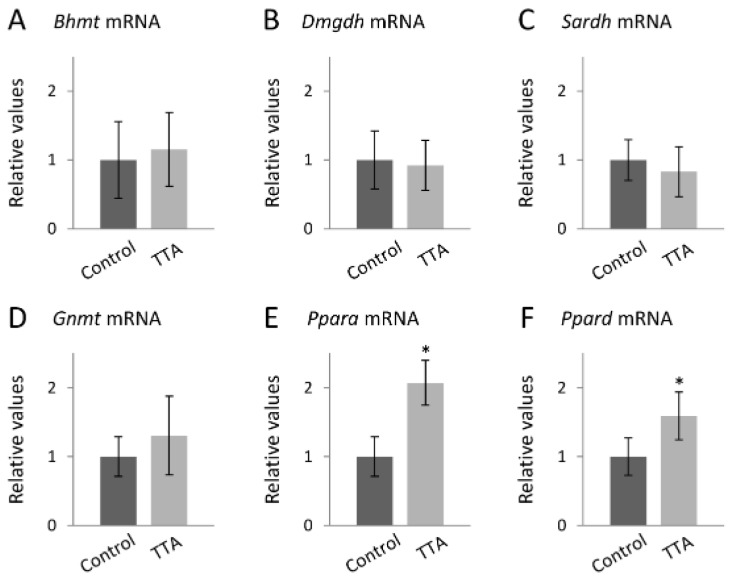
Gene expression in liver of rats receiving a high fat control diet or a high fat diet with additional TTA treatment. Male Wistar rats were treated with a high-fat diet with or without additional TTA treatment, *n* = 10 in each group. RNA was purified from frozen liver samples, and gene expression was quantified relative to a reference gene. The bars represent the relative gene expression of *Bhmt* (**A**); *Dmgdh* (**B**); *Sardh* (**C**); *Gnmt* (**D**); *Pparα* (**E**); and *Ppard* (**F**) compared to the Control group. Group differences are evaluated with *t*-test and Mann-Whitney U test, and * indicates *p* < 0.001. Bhmt indicates betaine-homocysteine methyltransferase; Dmgdh, dimethylglycine dehydrogenase; Gnmt, glycine N-methyltransferase; Ppar, peroxisome proliferator-activated receptor; Sardh, sarcosine dehydrogenase; TTA, tetradecylthioacetic acid.

The hepatic gene expression analyses showed no difference in the expression of *Bhmt* (Figure 4A), *Dmgdh* (Figure 4B), *Sardh* (Figure 4C) or *Gnmt* (Figure 4D). However, *Ppara* mRNA was increased 2.1-fold (*p* < 0.001) (Figure 4E) and *Ppard* mRNA was increased 1.5-fold (*p* < 0.001) (Figure 4F) in the TTA group.

## 4. Discussion

### 4.1. Principal Findings

This long-term, 50 weeks, animal study indicated that TTA treatment was associated with pronounced effects on the hepatic gene expression of PPARα and PPARβ/δ and on circulating concentrations of metabolites along the choline oxidation pathway and one-carbon metabolism as well as markers of B-vitamin status. The largest effect sizes were observed for plasma concentrations of DMG, NAM, mNAM, MMA and PL, which were all higher in the TTA group. Our results extend on previous findings by demonstrating that PPARs may also affect the plasma concentrations of metabolites in the choline oxidation and one-carbon metabolism pathways, as well as circulating levels of closely related B-vitamins.

### 4.2. Possible Mechanisms

#### 4.2.1. TTA Treatment and the Choline Oxidation Pathway

The particularly high concentration of DMG associated with TTA treatment, both in plasma and in urine, could be explained by TTA induced alterations in DMG production, catabolism, urinary excretion or a combination thereof. At least part of the association is probably explained by decreased catabolism of both DMG and sarcosine, as supported by the lower protein levels of DMGDH and SARDH previously reported in TTA-treated animals [25]. However, as the hepatic gene expression of *Dmgdh* and *Sardh* was not different between groups, this could be related to post-transcriptional regulation or other mechanisms such as limited availability of cofactors. Both DMGDH and SARDH are flavoproteins [37], and lower circulating concentrations of vitamin B2 as observed in this study may thus reduce DMG catabolism. Moreover, folate-dependent remethylation of Hcy by MS utilizes mTHF, which is produced from methylenetetrahydrofolate by methylenetetrahydrofolate reductase (MTHFR, EC 1.5.1.20), and the reaction depends on MS reductase (MSR, EC 1.16.1.8). Notably, both MTHFR and MSR are flavoproteins [38,39]. Accordingly, we observed markedly lower levels of folate in the TTA-treated rats, and as mTHF makes up the majority of circulating folate [40], this suggests reduced MTHFR flux. Hence, reduced MS flux due to lower concentrations of mTHF, may lead to a compensatory increase in BHMT-mediated remethylation, enhancing DMG production.

Data on systemic sarcosine may have shed further light on potential changes of metabolites downstream of DMG. Unfortunately, we were not able to determine sarcosine in plasma due to analytical interference from the EDTA in tubes used for blood sampling. However, urinary sarcosine tended to be higher in the TTA treated rats, indicating an increased glomerular filtration or decreased fractional tubular reabsorption secondary to a probable higher plasma concentration. This was further supported by the strong correlations between plasma and urinary levels of the closely related metabolites DMG and betaine. Sarcosine can be produced from glycine in the cell cytosol, via GNMT. As PPARα activation is suggested to inhibit flux through GNMT [13,24], decreased cellular sarcosine production from glycine may have contributed to the higher plasma concentrations of glycine and serine observed among the TTA treated rats. In line with this, a recent study found increased concentrations of glycine and serine after PPARα activation, and a metabolic tracer experiment revealed that increased rate of appearance into plasma, not decreased clearance or catabolism, was the main mechanism responsible for this observation [41].

There are several possible routes for glycine synthesis that may be affected by TTA. Increased production from sarcosine via SARDH is unlikely, due to the known inhibitory effect of PPARα activation on SARDH [13,25]. Moreover, glycine may be formed from serine, which can be derived through glycolysis [42], but as the glycolytic pathway is known to be inhibited by PPARα activity [15] this is also not a likely source. Another possible route of glycine synthesis is from threonine catabolism [42], which should be further explored as the plasma threonine concentration has previously been reported to be markedly higher among the TTA-treated rats [28]. Notably, both PPARα activation [43] and TTA treatment [28] are associated with increased synthesis of carnitine, which plays an essential role in fatty acid metabolism [44]. Because the production of each molecule of carnitine also yields one molecule of glycine [44], it is reasonable to suspect increased carnitine synthesis being a contributor to the elevated glycine concentrations.

#### 4.2.2. TTA Treatment and Vitamin B3

B3 vitamers are cofactors for a vast number of enzymatic redox reactions, such as the β-oxidation of fatty acids and substrate oxidation in Krebs cycle [45], as well as in the synthesis of carnitine [46]. In one-carbon metabolism, vitamin B3 is used as a reducing agent for both MTHFR and MSR [38,39], as well as in the conversion of choline to betaine [47]. The primary cofactor form of vitamin B3, nicotinamide adenine dinucleotide (phosphate) (NAD[P]), is formed from NAM and NA, and in the current study TTA treatment was associated with significantly higher concentration of NAM and also its breakdown metabolite mNAM. PPARα activation by WY14,643 was previously suggested to increase the production of NAM originating from the catabolism of tryptophan [13,26,48], and accordingly, such treatment has consistently been associated with elevated urinary concentrations of both NAM and mNAM [13,49,50]. Higher plasma NAM and mNAM observed in the TTA group may thus be due to PPARα-induced increased production, and may be related to increased requirements for vitamin B3 due to enhanced β-oxidation, a well-known PPARα effect [51].

#### 4.2.3. TTA Treatment and Vitamin B6

The transsulfuration pathway is activated by oxidative stress [52], and systemic vitamin B6 deficiency has previously been associated with both increased oxidative stress [53] and inflammation [54]. Of the B6 vitamers, only PL differed significantly between groups, with higher concentrations being observed in the TTA group. Although the most commonly used marker of vitamin B6 status is pyridoxal-5’-phosphate (PLP), total plasma B6-aldehyde (PL+PLP) is suggested as a direct measure of B6 status [55]. Higher B6-aldehyde, as indicated by the higher levels of PL, may thus represent improved B6 status, which is associated with lower inflammation and oxidative stress [53,54]. This is consistent with the anti-inflammatory and anti-oxidative effects previously seen by PPARα activation [14,15] and TTA treatment [18,56]. Notably, it has been demonstrated in cell studies that the gene expression of alkaline phosphatase (EC 3.1.3.1), the enzyme responsible for conversion of PLP to PL in plasma, is upregulated after PPARα activation [57]. This may explain why PL, and not PLP, was higher after TTA treatment. However, the production of PLP from pyridoxine and pyridoxamine is catalyzed by the flavoprotein pyridoxamine-pyridoxine 5-phosphate oxidase, and lower availability of vitamin B2 may limit PLP production via this route, possibly adding to the explanation why PLP is not increased [58].

#### 4.2.4. TTA Treatment and Vitamin B12 Status

In this study, higher concentrations of MMA were observed in both plasma and urine among the TTA treated rats. Plasma cobalamin, however, was unaffected, indicating a metabolic cobalamin deficiency not reflected by low circulating cobalamin levels. This is in accordance with the observation that serum cobalamin is often poorly correlated with clinical signs and the functional markers of B12 deficiency [59]. The intracellular processing of cobalamin is complex and involves several enzymes which, to our knowledge, have not been evaluated as candidate PPARα targets. The protein expression of methylmalonyl-CoA mutase (MUT, EC 5.4.99.2), which catalyzes the catabolism of methylmalonyl-CoA in the mitochondria, was reported to be elevated after TTA treatment [25]. This is not in agreement with the elevated plasma and urinary MMA observed in the present study, and may be a compensatory up regulation due to other TTA-induced metabolic alterations upstream of the MUT reaction. One potential mechanism could be inhibition of the methylmalonic aciduria combined with homocystinuria type C (MMACHC) protein. MMACHC is a flavoprotein responsible for making free cobalamin available for cofactor synthesis [60], and lower availability of vitamin B2 may thus reduce MMACHC function. Also, MMACHC is dependent on glutathione transferase activity [61], linking cobalamin metabolism to the transsulfuration pathway, which is a substantial source of cysteine for glutathione synthesis [62]. PPARα activation is known to inhibit the transcription of both enzymes in the transsulfuration pathway [13,63]. Elevated plasma cystathionine, observed after TTA treatment, has previously been associated with impaired transsulfuration due to B6-restriction [64]. However, B6 depletion was not followed by decreased glutathione synthesis, indicating that glutathione synthesis might primarily be regulated by other mechanisms [52]. The anti-inflammatory and anti-oxidative properties of TTA may result in decreased glutathione synthesis, which then might hamper MMACHC function. Together, lower availability of B2 and glutathione might reduce MMACHC function, leading to a functional cobalamin deficiency, a known effect of MMACHC defects [65]. In terms of MMA synthesis, the precursor for methylmalonyl-CoA is propionyl-CoA, derived from the catabolism of both odd-chained fatty acids and branched-chained amino acids. Increased oxidation of fatty acids is a well-known PPARα-effect, and it has been suggested that the catabolism of branched-chained amino acids is stimulated by PPARα activity [66], possibly facilitating increased MMA production.

### 4.3. Strengths and Limitations

The main strength of this study is its randomized and controlled long-term dietary intervention design. Extrapolation of the results to humans may, however, not be straightforward, as PPARα activation affects rodents differently and to a larger extent than humans [67,68]. We can also not exclude the possibility of PPAR-independent effects of TTA treatment influencing the results, thus limiting the interpretation of the observations simply being due to PPARα activation. The animals were sacrificed under non-fasting condition, which can be regarded a limitation of the assessment of metabolites. In humans, we have shown that DMG concentrations are higher among non-fasting individuals as compared to those with fasting samples [6]. Furthermore, blood and urinary concentrations of the various metabolites do not necessarily reflect their tissue concentrations, prompting careful interpretation in terms of metabolic flux [69]. It should also be acknowledged that the different B-vitamin cofactors are involved in a myriad of metabolic pathways not discussed herein, which could be of clinical interest related to PPAR activity. In terms of genes, it should be regarded a limitation that we only have analyzed the hepatic expression, and not the expression in other tissues which might be different.

### 4.4. Clinical Application

The involvement of PPARs in nutrient metabolism is well established. Thus, information on the activity of PPARs, and PPARα in particular, may be of future interest when considering tailored treatment or nutritional advice to the individual person. Metabolomics has been proposed as an important tool to understand PPARα function [50], and targeted metabolic profiling focusing on one-carbon metabolites may provide valuable information regarding PPARα activity. DMG, MMA and vitamin B3 metabolites could be promising targets for such metabolic profiling in humans.

## 5. Conclusions

We have demonstrated that long-term treatment with TTA is associated with altered plasma and urinary concentrations of metabolites related to one-carbon metabolism and B-vitamin status in rats. We did not observe any differences in the hepatic gene expression of genes related to the choline oxidation pathway, but the expression of PPARα was increased. Our findings should motivate further investigation into how these metabolic pathways are regulated, specifically by looking into the suggested role of PPARα and explore whether these metabolites may reflect hepatic PPARα activity.
